# Crystal structure and Hirshfeld surface analysis of 4-(2,6-di­chloro­benz­yl)-6-[(*E*)-2-phenyl­ethen­yl]pyridazin-3(2*H*)-one

**DOI:** 10.1107/S205698902001573X

**Published:** 2021-01-01

**Authors:** Said Daoui, Emine Berrin Cinar, Necmi Dege, Tarik Chelfi, Fouad El Kalai, Abdulmalik Abudunia, Khalid Karrouchi, Noureddine Benchat

**Affiliations:** a Laboratory of Applied Chemistry and Environment (LCAE), Faculty of Sciences, Mohamed I University, 60000 Oujda, Morocco; b Department of Physics, Faculty of Arts and Sciences, Ondokuz Mayıs University, Samsun, 55200, Turkey; c Department of Pharmacology, Faculty of Clinical Pharmacy, University of Medical and Applied Sciences, Yemen; d Laboratory of Analytical Chemistry and Bromatology, Faculty of Medicine and Pharmacy, Mohammed V University, Rabat, Morocco

**Keywords:** crystal structure, Hirshfeld surface analysis, pyridazine derivative, pyridazinone

## Abstract

The title pyridazinone derivative, C_19_H_14_Cl_2_N_2_O, an important pharmacophore with a wide variety of biological applications is not planar, the chloro­phenyl and pyridazinone rings being almost perpendicular, subtending a dihedral angle of 85.73 (11)°. The phenyl ring of the styryl group is coplanar with the pyridazinone ring [1.47 (12)°]. In the crystal, N—H⋯O hydrogen bonds form inversion dimers with an *R*_2_^2^(8) ring motif and C—H⋯Cl hydrogen bonds also occur. The roles of the inter­molecular inter­actions in the crystal packing were clarified using Hirshfeld surface analysis, and two-dimensional fingerprint plots indicate that the most important contributions to the crystal packing are from H⋯H (37.9%), C⋯H/H⋯C (18.7%), Cl⋯H/ H⋯Cl (16.4%) and Cl⋯C/C⋯Cl (6.7%) contacts.

## Abstract 2

In the title pyridazinone derivative, the chloro­phenyl and pyridazinone rings being almost perpendicular, while the phenyl ring of the styryl group is coplanar with the pyridazinone ring. In the crystal, N—H⋯O hydrogen bonds form inversion dimers with an 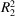
(8) ring motif and C—H⋯Cl hydrogen bonds also occur.

## Chemical context   

Pyridazines are an important family of six-membered aromatic heterocycles containing two nitro­gen atoms. Pyridazinone is an important pharmacophore possessing a wide range of biological activities including anti­tumor (Bouchmaa *et al.*, 2018 ▸, 2019 ▸), anti-inflammatory (Boukharsa *et al.*, 2018 ▸), anti­hypertensive (Siddiqui *et al.*, 2011 ▸), anti­depressant (Boukharsa *et al.*, 2016 ▸), anti-HIV (Livermore *et al.*, 1993 ▸), anti­histaminic (Tao *et al.* 2012 ▸), analgesic (Gökçe *et al.*, 2009 ▸) and anti­convulsant (Partap *et al.*, 2018 ▸) and is used in glucan synthase inhibitors (Zhou *et al.*, 2011 ▸) and herbicidal agents (Asif *et al.*, 2013 ▸). The chemistry of pyridazinones has been an inter­esting field of study for decades and this nitro­gen heterocycle has become a scaffold of choice for the development of potential drug candidates (Dubey *et al.*, 2015 ▸; Thakur *et al.*, 2010 ▸).
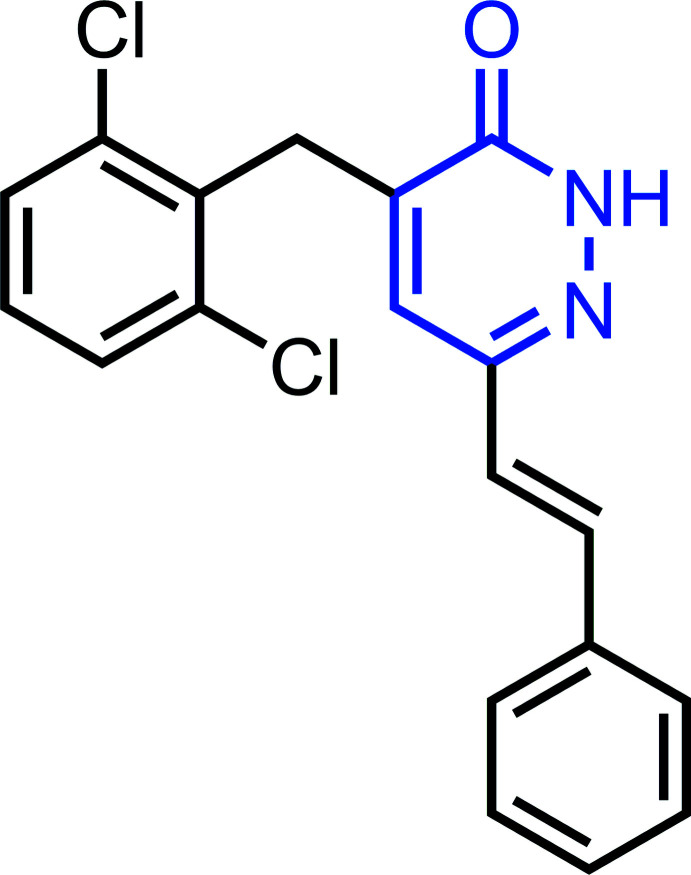
In a continuation of our studies towards the synthesis, mol­ecular structures, Hirshfeld surfaces analysis and DFT studies of new pyridazin-3(2*H*)-one derivatives (Daoui *et al.*, 2020 ▸, 2021 ▸; El Kalai *et al.*, 2021 ▸), we report herein the crystal structure and Hirshfeld surface analysis of 4-(2,6-di­chloro­benz­yl)-6-[(*E*)-2-phenyl­ethen­yl]pyridazin-3(2*H*)-one.

## Structural commentary   

**Figure 1:**
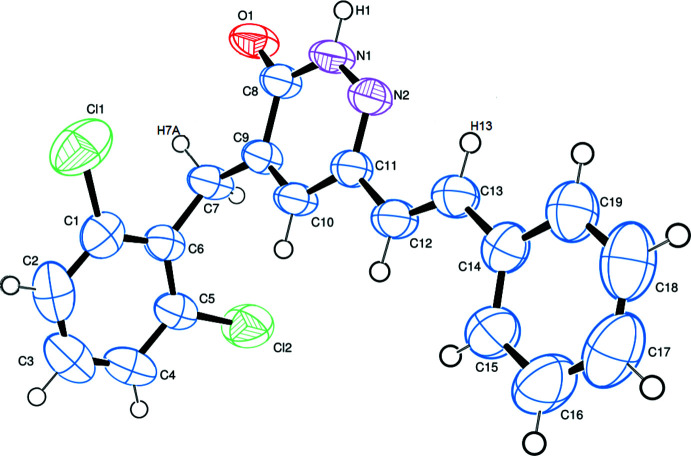
The mol­ecular structure of the title compound, with the atom labelling. Displacement ellipsoids are drawn at the 50% probability level.

The mol­ecular structure of the title compound is shown in Fig. 1 ▸. The C1–C6 phenyl ring and the pyridazinone ring (N1/N2/C8–C11) are almost perpendicular, subtending a dihedral angle of 85.73 (11)°. The C14–C19 phenyl ring of the styryl group is coplanar with the pyridazinone ring [1.47 (12)°]. The carbonyl group has a C8=O1 bond length of 1.236 (2) Å, and the C8—N1 and C11—N2 bond lengths in the pyridazine ring are 1.357 (3) and 1.305 (2) Å, respectively. The N1—N2 bond length is 1.344 (2) Å.

## Supra­molecular features   

**Figure 2:**
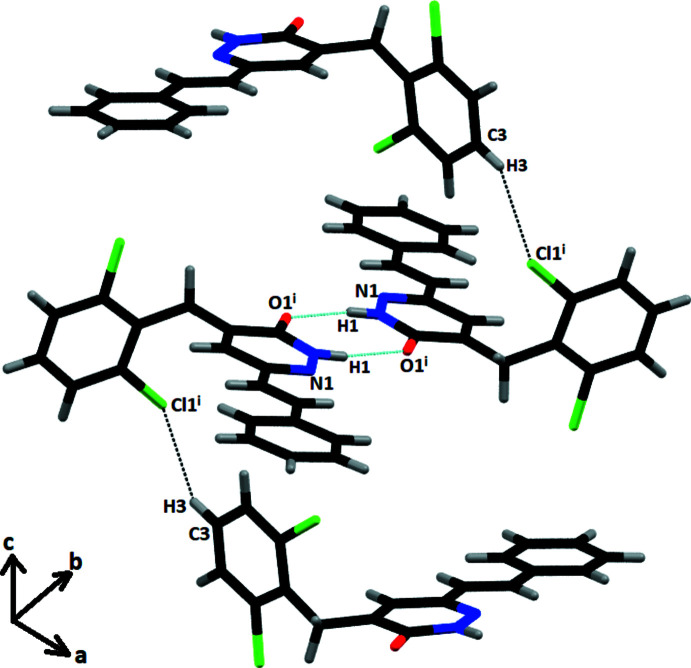
View of the crystal structure of the title compound. N—H⋯O hydrogen bonds are represented by red dashed lines and C—H⋯N and C—H⋯O inter­actions are shown as blue dashed lines.

**Figure 3:**
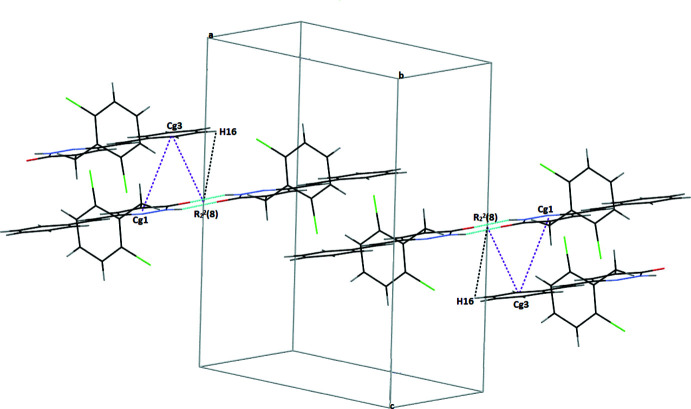
Packing diagram showing the inter­molecular inter­actions in the title compound (C—H⋯π inter­actions shown as black dashed lines and π–π inter­actions as purple dashed lines).

In the crystal, pairs of N—H⋯O hydrogen bonds form inversion dimers with an 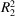
(8) ring motif (Table 1 ▸, Fig. 2 ▸). C3—H3⋯Cl1 hydrogen bonds are also observed. C—H⋯π inter­actions between the 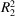
(8) dimer rings and H16 atoms [centroid-to-centroid distance of 3.501 (9) Å; length between dimer ring and C14–C19 ring = 3.569 (12) Å] also occur (Fig. 3 ▸). π–π inter­actions also occur with a centroid–centroid distance *Cg*1⋯*Cg*3(−*x* + 1, −*y* + 2, −*z* + 1) of 3.9107 (15) Å where *Cg*1 and *Cg3* are the centroids of the N1/N2/C8–C11 and C14–C19 rings, respectively (Fig. 3 ▸).

## Database survey   

A survey of the Cambridge Structural Database (CSD version 5.41, update of March 2020; Groom *et al.*, 2016 ▸) reveals six comparable pyridazine derivatives, 1-(6-benzoyl-2-phenyl-2,3-di­hydro­pyridazin-4-yl)ethanone 1-(4-benzoyl-2-phenyl-2,3-di­hydro­pyridazin-6-yl)ethanone (AQIKOB; Al-Awadi *et al.*, 2011 ▸), 4-(2′-chloro-6′-fluoro­phen­yl)-2,5-dioxo-8-phenyl-1,2,3,4,5,6-hexa­hydro­pyrido(2,3-*d*)pyridazine (BARQOA; Pita *et al.*, 2000 ▸), 4-[(2,6-di­chloro­phen­yl)meth­yl]-6-phenyl­pyridazin-3(2*H*)-one (BOBXEY; El Kali, Kansiz *et al.*, 2019 ▸), ethyl {5-[(3-chloro­phen­yl)meth­yl]-6-oxo-3-phenyl­pyridazin-1(6*H*)-yl}acetate (FODQUN; El Kalai, Baydere *et al.*, 2019 ▸), 4-benzyl-2-[2-(4-fluoro­phen­yl)-2-oxoeth­yl]-6-phenyl­pyrid­az­in-3(2*H*)-one (NOLDUQ; Daoui *et al.*, 2019 ▸) and 4-benzyl-6-*p*-tolyl­pyridazin-3(2*H*)-one (YOTVIN; Oubair *et al.*, 2009 ▸). Of these, BOBXEY, (II), is very similar to the title compound. The phenyl ring and the pyridazine ring are twisted with respect to each other, making a dihedral angle of 21.76 (18)° and the phenyl ring (C1–C6) of the benzyl group is inclined to the pyridazine ring by 79.61 (19)°. Relevant bond lengths in (II) are C17=O1 = 1.229 (5), C17—N2 = 1.388 (5) Å and C10—N1 =1.299 (4) Å. The N1—N2 bond lengths in (I) and (II) are virtually the same, with values of 1.348 (2) and 1.353 (4) Å, respectively. In the structure of YOTVIN, N—H⋯O bonds are also observed.

## Hirshfeld surface analysis   

**Figure 4:**
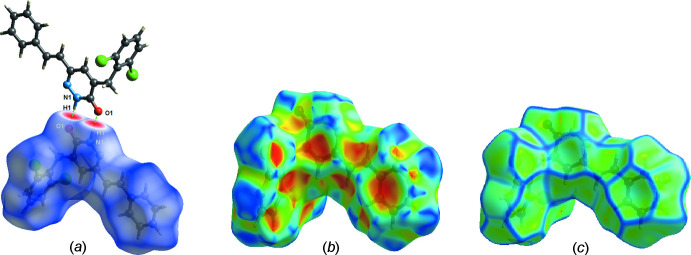
(*a*) Hirshfeld surface mapped over *d*_norm_ for visualizing the inter­molecular inter­actions of the title compound, (*b*) shape-index map and (*c*) curvedness map of the title mol­ecule.

**Figure 5:**
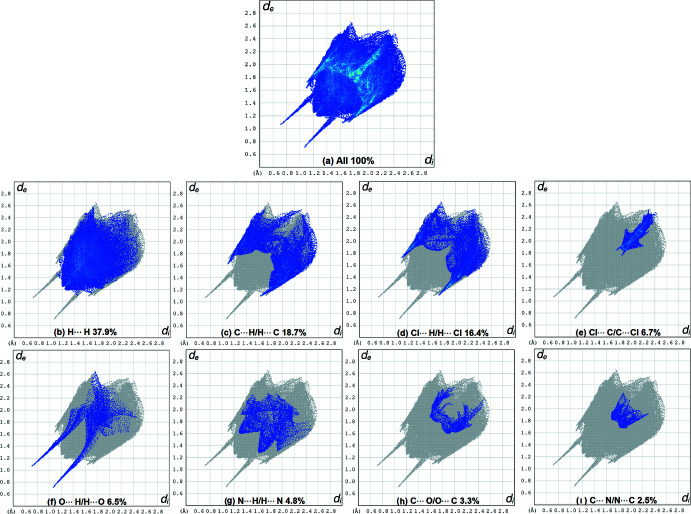
Two-dimensional fingerprint plots for the title compound showing the relative contributions of the atom pairs to the Hirshfeld surface.

**Figure 6:**
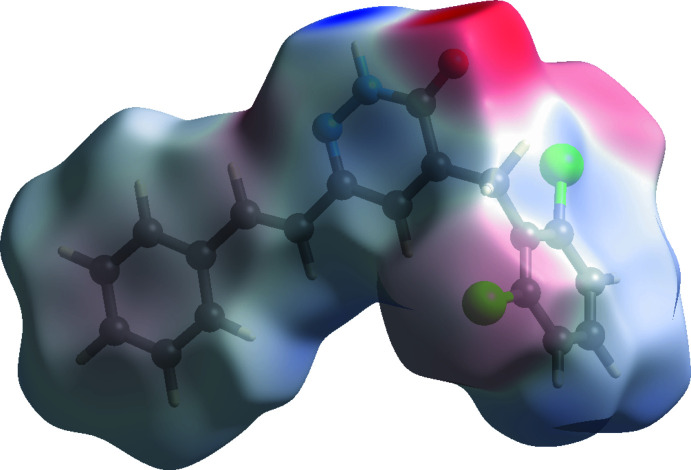
A view of the three-dimensional Hirshfeld surface of the title compound plotted over electrostatic potential.

A Hirshfeld surface (HS) study of the title compound was undertaken using *CrystalExplorer17.5* (Turner *et al.*, 2017 ▸) to visualize and study the inter­molecular contacts. The *d*_norm_ surface of the title compound is illustrated in Fig. 4 ▸ *a*. The shape-index, a tool for visualizing π–π stacking inter­actions by the presence of adjacent red and blue triangles is given in Fig. 4 ▸ *b* while Fig. 4 ▸ *c* shows the curvedness map of the title compound. The absence of prominent red and blue triangles in the shape-index map, as well as the absence of large green regions in the curvedness map, confirms that π–π and C—H⋯π interactions are weak. Fig. 5 ▸ shows fingerprint plots that qu­anti­tatively summarize the nature and type of inter­molecular contacts. The highest contribution to the Hirshfeld surface is from H⋯H contacts (Fig. 5 ▸ *b*). Other inter­actions and their respective contributions are C⋯H/H⋯C (18.7%), Cl⋯H/H⋯Cl (16.4%), Cl⋯C/C⋯Cl (6.7%), O⋯H/H⋯O (6.5%), N⋯H/H⋯N (4.8%), C⋯O/O⋯C (3.3%) and C⋯N/N⋯C (2.5%). The acceptor and donor atoms participating in the hydrogen bond appear as blue (donors) and red regions (acceptors) corresponding to positive and negative potential, respectively, in the HS mapped over the electrostatic potential, in the range −0.099–0.165 a.u., as shown in Fig. 6 ▸.

## Synthesis and crystallization   

To a solution of (*E*)-6-styryl-4,5-di­hydro­pyridazin-3(2*H*)-one (0.2 g, 1 mmol) and 2,6-di­chloro­benzaldehyde (0.175 g, 1 mmol) in 30 ml of ethanol, sodium ethano­ate (0.23 g, 2.8 mmol) was added. The mixture was refluxed for 3 h. The reaction mixture was cooled, diluted with cold water and acidified with concentrated hydro­chloric acid. The precipitate was filtered, washed with water, dried and recrystallized from ethanol. Colourless single-crystals were obtained by slow evaporation at room temperature.

## Refinement   

Crystal data, data collection and structure refinement details are summarized in Table 2 ▸. C-bound H atoms were positioned geometrically with C—H distances of 0.93–0.97 Å and refined as riding, with *U*_iso_(H) = 1.2*U*_eq_(C). The N-bound H atom was located in a difference-Fourier map and refined with N—H = 0.86 Å. 

## Supplementary Material

Crystal structure: contains datablock(s) I. DOI: 10.1107/S205698902001573X/dx2034sup1.cif

Structure factors: contains datablock(s) I. DOI: 10.1107/S205698902001573X/dx2034Isup2.hkl

Supporting information file. DOI: 10.1107/S205698902001573X/dx2034Isup3.cml

CCDC reference: 2047452

Additional supporting information:  crystallographic information; 3D view; checkCIF report

## Figures and Tables

**Table 1:** Hydrogen-bond geometry (Å, °)

*D*—H⋯*A*	*D*—H	H⋯*A*	*D*⋯*A*	*D*—H⋯*A*
N1—H1⋯O1^i^	0.86	1.92	2.772 (2)	171
C3—H3⋯Cl1^ii^	0.93	2.97	3.824 (3)	153
C7—H7*A*⋯O1	0.97	2.42	2.803 (2)	103
C13—H13⋯N2	0.93	2.51	2.845 (3)	101

Symmetry codes: (i) 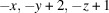; (ii) 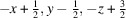.

**Table 2:** Experimental details

Crystal data	
Chemical formula	C_19_H_14_Cl_2_N_2_O
*M*_r_	357.22
Crystal system, space group	Monoclinic, *P*2_1_/*n*
Temperature (K)	296
*a*, *b*, *c* (Å)	10.1306 (5), 10.7019 (6), 15.7749 (7)
β (°)	97.715 (4)
*V* (Å^3^)	1694.78 (15)
*Z*	4
Radiation type	Mo *K*α
μ (mm^−1^)	0.39
Crystal size (mm)	0.72 × 0.47 × 0.13

Data collection	
Diffractometer	Stoe IPDS 2
Absorption correction	Integration (*X-RED32*; Stoe & Cie, 2002 ▸)
*T*_min_, *T*_max_	0.796, 0.937
No. of measured, independent and observed [*I* > 2σ(*I*)] reflections	20123, 5828, 2944
*R*_int_	0.047
(sin θ/λ)_max_ (Å^−1^)	0.746

Refinement	
*R*[*F* ^2^ > 2σ(*F* ^2^)], *wR*(*F* ^2^), *S*	0.064, 0.170, 1.01
No. of reflections	5828
No. of parameters	217
H-atom treatment	H-atom parameters constrained
Δρ_max_, Δρ_min_ (e Å^−3^)	0.33, −0.21

Computer programs: *X-AREA* and *X-RED32* (Stoe & Cie, 2002 ▸), *SHELXT2018/3* (Sheldrick, 2015*a* ▸), *SHELXL2018/3* (Sheldrick, 2015*b* ▸), *OLEX2* (Dolomanov *et al.*, 2009 ▸), *Mercury* (Macrae *et al.*, 2020 ▸), *WinGX* (Farrugia, 2012 ▸), *PLATON* (Spek, 2020 ▸) and *publCIF* (Westrip, 2010 ▸).

## supplementary crystallographic information

### Crystal data

**Table:**

C_19_H_14_Cl_2_N_2_O	*F*(000) = 736
*M**_r_* = 357.22	*D*_x_ = 1.400 Mg m^−^^3^
Monoclinic, *P*2_1_/*n*	Mo *K*α radiation, λ = 0.71073 Å
*a* = 10.1306 (5) Å	Cell parameters from 16480 reflections
*b* = 10.7019 (6) Å	θ = 1.9–32.4°
*c* = 15.7749 (7) Å	µ = 0.39 mm^−^^1^
β = 97.715 (4)°	*T* = 296 K
*V* = 1694.78 (15) Å^3^	Plate, colorless
*Z* = 4	0.72 × 0.47 × 0.13 mm

### Data collection

**Table:**

Stoe IPDS 2 diffractometer	5828 independent reflections
Radiation source: sealed X-ray tube, 12 x 0.4 mm long-fine focus	2944 reflections with *I* > 2σ(*I*)
Plane graphite monochromator	*R*_int_ = 0.047
Detector resolution: 6.67 pixels mm^-1^	θ_max_ = 32.0°, θ_min_ = 2.3°
rotation method scans	*h* = −12→15
Absorption correction: integration (X-RED32; Stoe & Cie, 2002)	*k* = −15→15
*T*_min_ = 0.796, *T*_max_ = 0.937	*l* = −23→23
20123 measured reflections	

### Refinement

**Table:**

Refinement on *F*^2^	Primary atom site location: structure-invariant direct methods
Least-squares matrix: full	Secondary atom site location: difference Fourier map
*R*[*F*^2^ > 2σ(*F*^2^)] = 0.064	Hydrogen site location: inferred from neighbouring sites
*wR*(*F*^2^) = 0.170	H-atom parameters constrained
*S* = 1.01	*w* = 1/[σ^2^(*F*_o_^2^) + (0.0652*P*)^2^ + 0.3156*P*] where *P* = (*F*_o_^2^ + 2*F*_c_^2^)/3
5828 reflections	(Δ/σ)_max_ < 0.001
217 parameters	Δρ_max_ = 0.33 e Å^−^^3^
0 restraints	Δρ_min_ = −0.21 e Å^−^^3^

### Special details

**Table:**

Geometry. All esds (except the esd in the dihedral angle between two l.s. planes) are estimated using the full covariance matrix. The cell esds are taken into account individually in the estimation of esds in distances, angles and torsion angles; correlations between esds in cell parameters are only used when they are defined by crystal symmetry. An approximate (isotropic) treatment of cell esds is used for estimating esds involving l.s. planes.

### Fractional atomic coordinates and isotropic or equivalent isotropic displacement parameters (Å^2^)

**Table:**

	*x*	*y*	*z*	*U*_iso_*/*U*_eq_	
Cl2	0.32888 (9)	0.44720 (8)	0.41784 (5)	0.1022 (3)	
Cl1	0.06171 (9)	0.57454 (9)	0.67843 (6)	0.1101 (3)	
O1	−0.00667 (14)	0.83981 (14)	0.49196 (12)	0.0698 (4)	
N2	0.30239 (16)	0.97613 (15)	0.57214 (12)	0.0561 (4)	
N1	0.17344 (16)	0.95588 (15)	0.54273 (13)	0.0582 (4)	
H1	0.1229	1.0208	0.5381	0.070*	
C6	0.19680 (18)	0.50436 (17)	0.55109 (14)	0.0520 (5)	
C11	0.37910 (19)	0.87772 (18)	0.58104 (13)	0.0508 (4)	
C9	0.2000 (2)	0.73781 (17)	0.53008 (13)	0.0508 (4)	
C8	0.11329 (19)	0.84552 (18)	0.51939 (14)	0.0536 (5)	
C10	0.32911 (19)	0.75621 (18)	0.56121 (14)	0.0533 (5)	
H10	0.3862	0.6880	0.5698	0.064*	
C12	0.5217 (2)	0.8968 (2)	0.61209 (14)	0.0568 (5)	
H12	0.5752	0.8261	0.6204	0.068*	
C7	0.1371 (2)	0.61526 (18)	0.50303 (16)	0.0609 (6)	
H7A	0.0433	0.6192	0.5095	0.073*	
H7B	0.1431	0.6031	0.4427	0.073*	
C14	0.7197 (2)	1.0298 (2)	0.66024 (15)	0.0615 (5)	
C13	0.5792 (2)	1.0060 (2)	0.62890 (15)	0.0607 (5)	
H13	0.5245	1.0759	0.6199	0.073*	
C1	0.1675 (2)	0.4752 (2)	0.63198 (16)	0.0650 (6)	
C5	0.2807 (2)	0.4200 (2)	0.51757 (16)	0.0631 (6)	
C19	0.7618 (3)	1.1510 (3)	0.67726 (17)	0.0761 (7)	
H19	0.7004	1.2159	0.6691	0.091*	
C2	0.2149 (3)	0.3694 (3)	0.67623 (18)	0.0840 (8)	
H2	0.1934	0.3533	0.7307	0.101*	
C15	0.8136 (2)	0.9359 (3)	0.67285 (19)	0.0776 (7)	
H15	0.7882	0.8536	0.6611	0.093*	
C4	0.3270 (3)	0.3132 (2)	0.5602 (2)	0.0831 (8)	
H4	0.3810	0.2579	0.5351	0.100*	
C3	0.2937 (3)	0.2891 (3)	0.6385 (2)	0.0897 (9)	
H3	0.3249	0.2168	0.6672	0.108*	
C18	0.8940 (3)	1.1772 (3)	0.7063 (2)	0.0951 (9)	
H18	0.9209	1.2595	0.7168	0.114*	
C16	0.9453 (3)	0.9629 (4)	0.7027 (2)	0.0969 (9)	
H16	1.0072	0.8984	0.7115	0.116*	
C17	0.9850 (3)	1.0827 (4)	0.7194 (2)	0.1007 (10)	
H17	1.0736	1.1002	0.7397	0.121*	

### Atomic displacement parameters (Å^2^)

**Table:**

	*U*^11^	*U*^22^	*U*^33^	*U*^12^	*U*^13^	*U*^23^
Cl2	0.1168 (6)	0.1083 (6)	0.0881 (5)	0.0366 (5)	0.0379 (5)	0.0082 (4)
Cl1	0.1042 (6)	0.1263 (7)	0.1069 (6)	−0.0057 (5)	0.0406 (5)	−0.0446 (5)
O1	0.0467 (8)	0.0506 (8)	0.1070 (13)	0.0102 (6)	−0.0080 (8)	−0.0061 (8)
N2	0.0479 (9)	0.0427 (9)	0.0767 (12)	0.0047 (7)	0.0040 (8)	−0.0020 (8)
N1	0.0459 (9)	0.0409 (8)	0.0860 (13)	0.0104 (7)	0.0017 (8)	−0.0034 (8)
C6	0.0467 (9)	0.0412 (9)	0.0653 (12)	−0.0024 (8)	−0.0024 (9)	−0.0062 (9)
C11	0.0469 (10)	0.0460 (10)	0.0587 (12)	0.0051 (8)	0.0047 (9)	0.0004 (9)
C9	0.0506 (10)	0.0406 (9)	0.0597 (12)	0.0066 (8)	0.0019 (9)	−0.0011 (8)
C8	0.0486 (10)	0.0434 (10)	0.0671 (13)	0.0070 (8)	0.0014 (9)	−0.0014 (9)
C10	0.0488 (11)	0.0415 (10)	0.0682 (13)	0.0114 (8)	0.0029 (9)	−0.0003 (9)
C12	0.0488 (10)	0.0493 (11)	0.0708 (14)	0.0067 (8)	0.0027 (9)	−0.0009 (9)
C7	0.0519 (11)	0.0451 (10)	0.0813 (15)	0.0073 (9)	−0.0074 (10)	−0.0068 (10)
C14	0.0553 (11)	0.0673 (14)	0.0618 (13)	−0.0049 (10)	0.0073 (10)	−0.0002 (11)
C13	0.0526 (11)	0.0545 (12)	0.0740 (14)	0.0054 (10)	0.0049 (10)	0.0029 (10)
C1	0.0605 (12)	0.0645 (13)	0.0692 (14)	−0.0135 (10)	0.0054 (11)	−0.0135 (11)
C5	0.0652 (13)	0.0512 (11)	0.0716 (14)	0.0087 (10)	0.0042 (11)	−0.0006 (10)
C19	0.0754 (16)	0.0741 (16)	0.0785 (16)	−0.0148 (13)	0.0092 (13)	−0.0052 (13)
C2	0.095 (2)	0.0852 (19)	0.0684 (16)	−0.0303 (16)	−0.0025 (15)	0.0149 (14)
C15	0.0567 (13)	0.0771 (16)	0.0962 (19)	−0.0020 (12)	−0.0002 (12)	0.0039 (14)
C4	0.0901 (18)	0.0569 (14)	0.099 (2)	0.0237 (13)	0.0024 (16)	0.0022 (14)
C3	0.105 (2)	0.0610 (16)	0.097 (2)	0.0033 (15)	−0.0089 (18)	0.0160 (15)
C18	0.098 (2)	0.103 (2)	0.0835 (19)	−0.043 (2)	0.0090 (16)	−0.0134 (17)
C16	0.0554 (14)	0.125 (3)	0.106 (2)	0.0002 (16)	−0.0032 (14)	0.010 (2)
C17	0.0643 (17)	0.141 (3)	0.093 (2)	−0.031 (2)	−0.0051 (15)	−0.002 (2)

### Geometric parameters (Å, º)

**Table:**

Cl2—C5	1.733 (3)	C14—C15	1.379 (3)
Cl1—C1	1.740 (3)	C14—C19	1.380 (3)
O1—C8	1.236 (2)	C14—C13	1.465 (3)
N2—C11	1.305 (2)	C13—H13	0.9300
N2—N1	1.344 (2)	C1—C2	1.382 (4)
N1—C8	1.357 (3)	C5—C4	1.376 (3)
N1—H1	0.8600	C19—C18	1.385 (4)
C6—C1	1.384 (3)	C19—H19	0.9300
C6—C5	1.392 (3)	C2—C3	1.362 (4)
C6—C7	1.492 (3)	C2—H2	0.9300
C11—C10	1.415 (3)	C15—C16	1.384 (4)
C11—C12	1.476 (3)	C15—H15	0.9300
C9—C10	1.349 (3)	C4—C3	1.350 (4)
C9—C8	1.445 (3)	C4—H4	0.9300
C9—C7	1.495 (3)	C3—H3	0.9300
C10—H10	0.9300	C18—C17	1.366 (5)
C12—C13	1.317 (3)	C18—H18	0.9300
C12—H12	0.9300	C16—C17	1.358 (5)
C7—H7A	0.9700	C16—H16	0.9300
C7—H7B	0.9700	C17—H17	0.9300
			
C11—N2—N1	116.38 (17)	C12—C13—H13	116.4
N2—N1—C8	127.90 (16)	C14—C13—H13	116.4
N2—N1—H1	116.1	C2—C1—C6	123.2 (2)
C8—N1—H1	116.1	C2—C1—Cl1	118.7 (2)
C1—C6—C5	114.9 (2)	C6—C1—Cl1	118.08 (19)
C1—C6—C7	121.7 (2)	C4—C5—C6	122.6 (2)
C5—C6—C7	123.3 (2)	C4—C5—Cl2	117.6 (2)
N2—C11—C10	121.83 (18)	C6—C5—Cl2	119.75 (17)
N2—C11—C12	117.79 (18)	C14—C19—C18	121.0 (3)
C10—C11—C12	120.38 (17)	C14—C19—H19	119.5
C10—C9—C8	118.07 (18)	C18—C19—H19	119.5
C10—C9—C7	125.98 (17)	C3—C2—C1	118.7 (3)
C8—C9—C7	115.93 (17)	C3—C2—H2	120.6
O1—C8—N1	121.52 (17)	C1—C2—H2	120.6
O1—C8—C9	123.72 (18)	C14—C15—C16	120.8 (3)
N1—C8—C9	114.76 (17)	C14—C15—H15	119.6
C9—C10—C11	121.03 (17)	C16—C15—H15	119.6
C9—C10—H10	119.5	C3—C4—C5	119.7 (3)
C11—C10—H10	119.5	C3—C4—H4	120.2
C13—C12—C11	125.18 (19)	C5—C4—H4	120.2
C13—C12—H12	117.4	C4—C3—C2	120.9 (3)
C11—C12—H12	117.4	C4—C3—H3	119.6
C6—C7—C9	115.12 (17)	C2—C3—H3	119.6
C6—C7—H7A	108.5	C17—C18—C19	120.2 (3)
C9—C7—H7A	108.5	C17—C18—H18	119.9
C6—C7—H7B	108.5	C19—C18—H18	119.9
C9—C7—H7B	108.5	C17—C16—C15	120.6 (3)
H7A—C7—H7B	107.5	C17—C16—H16	119.7
C15—C14—C19	117.8 (2)	C15—C16—H16	119.7
C15—C14—C13	122.9 (2)	C16—C17—C18	119.5 (3)
C19—C14—C13	119.3 (2)	C16—C17—H17	120.2
C12—C13—C14	127.2 (2)	C18—C17—H17	120.2
			
C11—N2—N1—C8	−1.5 (3)	C5—C6—C1—C2	−1.3 (3)
N1—N2—C11—C10	−0.3 (3)	C7—C6—C1—C2	176.1 (2)
N1—N2—C11—C12	179.18 (19)	C5—C6—C1—Cl1	−179.26 (16)
N2—N1—C8—O1	−178.9 (2)	C7—C6—C1—Cl1	−1.8 (3)
N2—N1—C8—C9	1.7 (3)	C1—C6—C5—C4	2.4 (3)
C10—C9—C8—O1	−179.6 (2)	C7—C6—C5—C4	−175.0 (2)
C7—C9—C8—O1	1.9 (3)	C1—C6—C5—Cl2	−178.40 (17)
C10—C9—C8—N1	−0.1 (3)	C7—C6—C5—Cl2	4.2 (3)
C7—C9—C8—N1	−178.7 (2)	C15—C14—C19—C18	−0.2 (4)
C8—C9—C10—C11	−1.4 (3)	C13—C14—C19—C18	−179.5 (2)
C7—C9—C10—C11	177.0 (2)	C6—C1—C2—C3	−0.4 (4)
N2—C11—C10—C9	1.7 (3)	Cl1—C1—C2—C3	177.5 (2)
C12—C11—C10—C9	−177.8 (2)	C19—C14—C15—C16	0.9 (4)
N2—C11—C12—C13	−2.5 (3)	C13—C14—C15—C16	−179.9 (3)
C10—C11—C12—C13	177.0 (2)	C6—C5—C4—C3	−1.7 (4)
C1—C6—C7—C9	78.6 (3)	Cl2—C5—C4—C3	179.0 (2)
C5—C6—C7—C9	−104.2 (2)	C5—C4—C3—C2	−0.2 (5)
C10—C9—C7—C6	31.4 (3)	C1—C2—C3—C4	1.2 (4)
C8—C9—C7—C6	−150.2 (2)	C14—C19—C18—C17	−0.7 (4)
C11—C12—C13—C14	179.6 (2)	C14—C15—C16—C17	−0.7 (5)
C15—C14—C13—C12	3.0 (4)	C15—C16—C17—C18	−0.2 (5)
C19—C14—C13—C12	−177.8 (2)	C19—C18—C17—C16	0.9 (5)

### Hydrogen-bond geometry (Å, º)

**Table:**

*D*—H···*A*	*D*—H	H···*A*	*D*···*A*	*D*—H···*A*
N1—H1···O1^i^	0.86	1.92	2.772 (2)	171
C3—H3···Cl1^ii^	0.93	2.97	3.824 (3)	153

Symmetry codes: (i) −*x*, −*y*+2, −*z*+1; (ii) −*x*+1/2, *y*−1/2, −*z*+3/2.
